# The clinical characteristics and therapeutic outcomes of adult patients with community-acquired spontaneous bacterial meningitis with a fulminant clinical course in Taiwan

**DOI:** 10.1186/s12879-023-08857-x

**Published:** 2023-12-06

**Authors:** Chia-Yi Lien, Chun-Chih Chien, Cheng-Hsien Lu, Wen-Neng Chang

**Affiliations:** 1 Department of Neurology, Kaohsiung Chang Gung Memorial Hospital, Chang Gung University College of Medicine, 123 Ta-Pei Road, Niao-Sung Section, Kaohsiung, Taiwan; 2 Department of laboratory medicine, Kaohsiung Chang Gung Memorial Hospital, Chang Gung University College of Medicine, Kaohsiung, Taiwan

**Keywords:** Bacterial Meningitis, Community-acquired, Spontaneous, End-stage renal Disease, High mortality

## Abstract

**Purpose:**

To examine the clinical characteristics of adult patients with community-acquired spontaneous bacterial meningitis (CASBM) with a fulminant clinical course.

**Materials and methods:**

The clinical features and therapeutic outcomes of 127 adult CASBM patients were analyzed. The patients were divided into two groups as those with and without a fulminant clinical course. Fulminant clinical course was defined as meningitis presenting initially with marked consciousness disturbance (Glasgow Coma Scale score < 8) or a rapid deterioration in consciousness level within 48 h of hospitalization.

**Results:**

Among the 127 enrolled patients, 69 had a fulminant clinical course (47 men and 22 women) and 58 did not. The patients with a fulminant clinical course had a significantly higher incidence of end-stage renal disease (ESRD), severe clinical manifestations and higher mortality rate, and the survivors had significantly worse therapeutic outcomes. *Klebsiella* (*K.*) *pneumoniae* (50 strains) was the most important pathogen for the development of a fulminant clinical course, and all strains were susceptible to ceftriaxone and ceftazidime. With treatment, 50.7% (35/69) of the patients with a fulminant clinical course died, and the presence of *K. pneumoniae* infection was significant prognostic factor.

**Conclusions:**

The presence of ESRD, initial presentation of altered consciousness, septic shock, seizures and CSF total protein level and *K. pneumoniae* infection were significantly associated with a fulminant clinical course of adult CASBM, and patients with this specific infectious syndrome had high mortality and morbidity rates. The presence of *K. pneumoniae* infection is a significant prognostic factor.

## Introduction

In recent decades, the epidemiologic trend of adult bacterial meningitis (ABM), a serious infectious disease of the central nervous system (CNS), has changed gradually [1], and the main changes included an increasing incidence of post-neurosurgical meningitis and a decreasing incidence of *Streptococcus pnemoniae* meningitis. The clinical course of bacterial meningitis is usually acute, but if the evolution of consciousness level is taken into consideration, its clinical course may range from hyper-acute to chronic [1–4]. In recent years, through the introduction of advanced management, the mortality rate of ABM in Taiwan has decreased to 25.5% [1, 5], however this figure of mortality and the rate of morbidity are still high [1]. *K. pneumoniae* is the most important pathogen of spontaneous bacterial meningitis, and we have reported this specific finding in serial published studies [6–9], and most of the patients with *K. pneumoniae* infection have severe medical comorbidity including diabetes mellitus and severe liver disease. Clinically, a group of adult patients with community-acquired spontaneous bacterial meningitis (CASBM) with a rapid evolution of clinical course from symptom onset to marked consciousness disturbance has been reported [1, 4]. In the study of R. Muralidharan et al. [4], they defined the fulminant bacterial meningitis as the meningitis presenting initially with marked consciousness disturbance [Glasgow Coma Scale score < 8] or a rapid deterioration in consciousness level within 48 h of the hospitalization. However, the detailed clinical characteristics of this specific group of patients have not previously been investigated thoroughly. Therefore, in this study, we analyzed the clinical characteristics and therapeutic outcomes of 69 adult CASBM patients with a fulminant course in order to better delineate this specific group ABM patients.

## Materials and methods

### Subjects

**Fig. 1:**
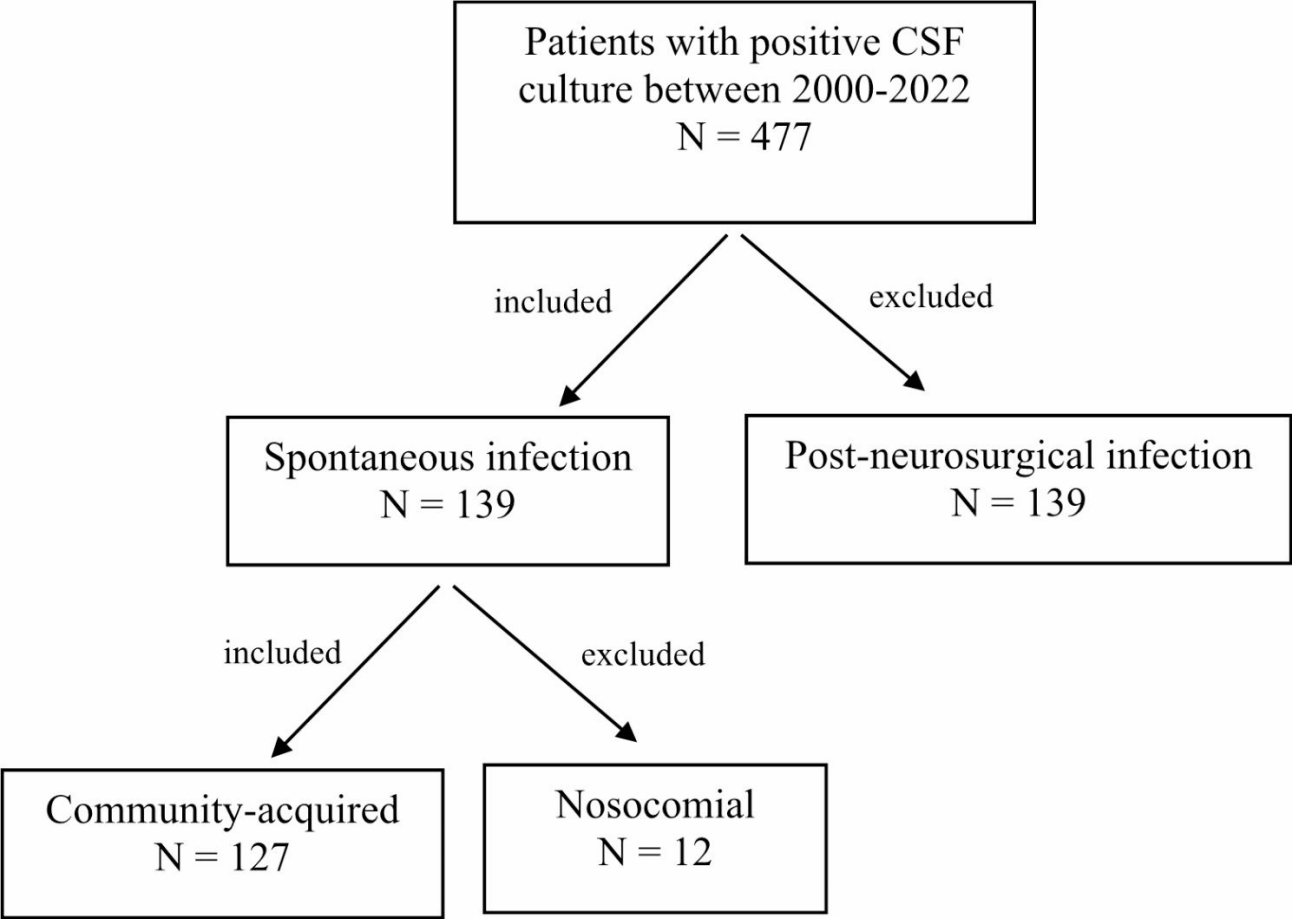
Diagram of participant enrollment Abbreviations: CSF: cerebrospinal fluid; N: number

We retrospectively reviewed the medical records of including microbiological records of cerebrospinal fluid (CSF), blood cultures, laboratory data, medical records and outcomes of adult patients (≥ 18 years) diagnosed with ABM and we enrolled patients with culture-proven bacterial meningitis admitted to Chang Gung Memorial Hospital (CGMH) – Kaohsiung, a 2680-bed acute-care teaching hospital providing both primary and tertiary care, over a period of 23 years (January 2000 to December 2022). During the study period, 447 culture-proven patients with ABM were identified, of whom 139 belonged to spontaneous infections and the other 308, post-neurosurgical form. Among these 139 patients, 12 had nosocomial infections and the other 127 belonged to CASBM. We excluded patients with nosocomial infection and post-neurosurgical infection and only the clinical and laboratory data of the 127 CASBM patients were enrolled in this study for analysis (Fig. 1). This study was approved by the hospital’s Ethics Committee (IRB No: 202101827B0).

### Diagnostic methods

In this study, the criteria for a definite diagnosis of bacterial meningitis were: (1) a positive CSF culture for bacterial pathogen(s); (2) clinical features of meningitis; and (3) purulent CSF features [1]. Patients who had not been admitted to the hospital, demonstrated no clear distinctive disease characteristics, and had not undergone any invasive procedures were classified as having CASBM [1]. Community-acquired meningitis was defined as positive bacterial infection presented when the patient was admitted to hospital or clinical evidence of an infection less than 48 h after the admission, or in out-hospital patients who developed the clinical evidence of meningitis in more than one month after prior hospitalization, especially those with a major surgical procedure including neurosurgical procedure or more than one month after the discharge from nursing home) [1, 10]. Patients were considered to have “mixed bacterial meningitis” if at least two bacterial organisms were isolated from the initial CSF culture [1]. A fulminant clinical course of the CASBM patients was defined as meningitis presenting initially with marked consciousness disturbance [Glasgow Coma Scale score < 8] or a rapid deterioration in consciousness level within 48 h of the hospitalization [4]. Bacteremia was defined as multiple blood cultures growing the same bacterial pathogen. Immunocompromised state was defined as primary immunodeficiency disorders [11] and secondary immunodeficiency state for patients with cancer, liver cirrhosis, end-stage renal disease (ESRD), long-term therapy with immunosuppressive agents for an overactive immune system or steroids for certain diseases, patients who had undergone organ transplantation and were receiving anti-rejection agent therapy, and patients with malnutrition [12]. In our hospital and most hospitals in Taiwan, the intravenous administration of penicillin G or vancomycin combined with a 3rd generation cephalosporin (ceftriaxone, ceftazidime) were used as the initial empiric antibiotics for treating adult patients with clinical evidence of bacterial meningitis. Further antibiotic treatment was then adjusted according to the results of pathogen identification and antibiotic susceptibility tests. The appropriateness of antibiotics use was defined as (1) administration of right empirical regimens as soon as possible upon the patients’ arrival of hospital, (2) adequate duration of antibiotics therapy as 3–4 weeks and adjustment of the therapy duration according to patients’ clinical condition and image finding and (3) adjustment antibiotics as soon as possible according to the susceptibility result from once the CSF culture result available [1, 4, 13, 14].

### Statistical analysis

For prognostic analysis, mortality and mRS [15] were used to evaluate the therapeutic results, and the patients were divided into two groups: those with good outcomes (mRS score = 0–2) and those with poor outcomes (mRS score ≥ 3). Initially, we analyzed all data using univariate logistic regression. For categorical variables related to fulminant bacterial meningitis and prognosis analysis, data including sex, underlying conditions, clinical manifestations, and therapeutic outcomes were analyzed using the *x*^2^-test; while differences in continuous variables such as age between the two groups and CSF data were analyzed using the Student’s t-test. Continuous data were expressed as the means ± standard deviations or medians (interquartile ranges), and the variables that were not normally distributed were logarithmically transformed to improve normality, and then compared using the independent t-test. Significant relationships among variables and the two patient groups were analyzed using stepwise multiple logistic regression analysis adjusted for other potential confounding factors. Variables with zero cell counts were eliminated from the logistic analysis, and only variables with statistical significance (*p* < 0.05) were included in the final model. All analyses were conducted using SSPS software version 22.0.

## Results

### Demographic data and clinical comparisons

**Fig. 2:**
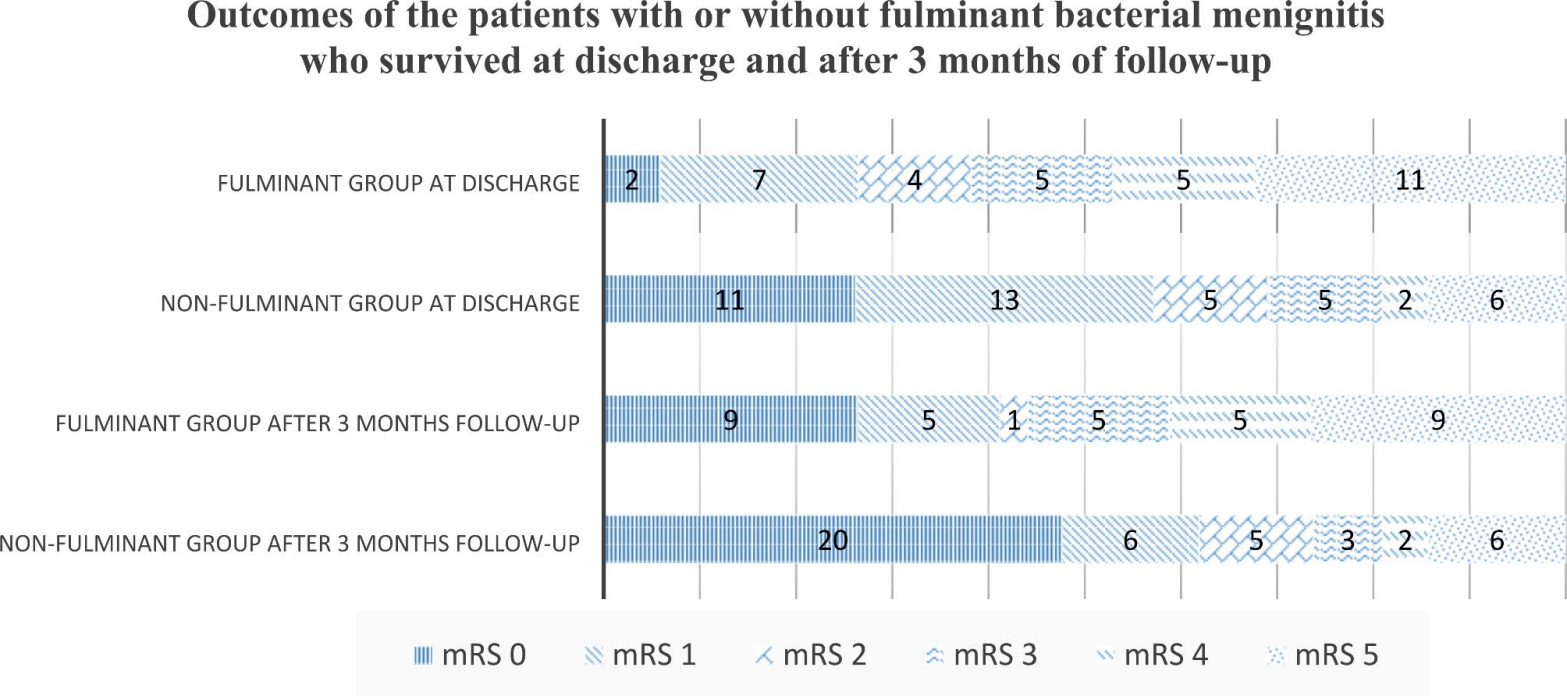
Distribution of the modified Rankin Scale scores of the survivor. mRS = modified Rankin scales

The demographic data, clinical features and therapeutic outcomes of the 127 enrolled CASBM patients are listed in Table 1. Among the 127 patients, 69 (47 men and 22 women, age range 19–83 years, median 59 years) had a fulminant clinical course (fulminant group), and 58 did not (non-fulminant group). The leading underlying conditions in the 69 patients in the fulminant group were immunocompromised state (41), diabetes mellitus (DM, 34), liver cirrhosis (16), alcoholism (15) and end-stage renal disease (ESRD, 10). Of the initial manifestations of the fulminant group, altered consciousness (63), fever (59), seizures (32) and septic shock (28) were the leading clinical features. Bacteremia was found in 37 of the 69 patients in the fulminant group. Compared to the non-fulminant group (Table 1), the presence of ESRD and bacteremia, initial presentation of altered consciousness, seizures, septic shock, diabetic ketoacidosis/hyperosmolar hyperglycemic state (DKA/HHS), and CSF total protein level were potentially different. However, after multiple logistic regression analysis, only the presence of septic shock was significantly difference. Although it did not reach the significance of statistical analysis, Table 1 also shows that the fulminant group had a significantly higher mortality rate (50.7% vs. 27.6%), and the survivors also had a significantly worse prognosis at discharge and at 3 months after discharge. The distribution of the mRS scores of the survivors is shown in Fig. 2.

**Table 1:** The clinical and laboratory features of the 127 community-acquired spontaneous adult bacterial meningitis patients with or without a fulminant clinical course

Characteristic	With fulminant course N = 69 (%)	With non-fulminant course N = 58 (%)	OR^a^	95% CI^a^	*p*-value	OR^b^	95% CI^b^	Adjusted *p*-value
Age	58.36 ± 15.36	56.47 ± 15.03			0.485			
Gender								
Male	47 (68.1)	41 (70.7)	1.129	0.529–2.411	0.754			
Female	22 (31.9)	17 (29.3)						
Outcome								
In-hospital mortality								
<72 h	8 (21.6)	0 (0.0)	1.276	1.078–1.511	0.038*	-	-	0.999
>72 h	13 (35.1)	0 (0.0)	1.541	1.217–1.953	0.005*	-	-	0.999
Discharge mRS								
At discharge	4.57 ± 1.90	2.97 ± 2.39			< 0.001*	-	-	0.999
Three months later	4.30 ± 2.24	2.74 ± 2.57			< 0.001*	-	-	0.999
Mortality	35 (50.7)	16 (27.6)	1.839	1.141–2.964	0.008*	-	-	0.583
Underlying condition								
Diabetes mellitus	34 (49.3)	22 (37.9)	1.299	0.865–1.952	0.200			
Liver cirrhosis	16 (23.2)	7 (12.1)	1.921	0.849–4.348	0.105			
Alcoholism	15 (21.7)	6 (10.3)	2.101	0.872–5.066	0.085			
Systemic malignancy	9 (13.0)	13 (22.4)	0.582	0.268–1.263	0.165			
Intracranial hemorrhage	7 (10.1)	11 (19.0)	0.535	0.222–1.291	0.156			
End-stage renal disease	10 (14.5)	1 (1.7)	8.406	1.109–63.732	0.011*	12.474	0.740–210.300	0.080
Substance abuser	3 (4.3)	2 (3.4)	1.261	0.218–7.291	0.795			
Brain tumor	1 (1.4)	3 (5.2)	0.280	0.030–2.622	0.231			
Immunocompromised state	41 (59.4)	26 (44.8)	1.326	0.938–1.873	0.101			
Clinical manifestations								
Fever	59 (85.5)	51 (87.9)	0.972	0.849–1.114	0.689			
Initial altered consciousness	63 (91.3)	37 (63.8)	1.431	1.164–1.761	< 0.001*	8.068	0.392-166.198	0.176
Seizure	32 (46.4)	9 (15.5)	2.989	1.557–5.736	< 0.001*	2.879	0.294–28.165	0.363
Septic shock	28 (40.6)	3 (5.2)	7.845	2.513–24.490	< 0.001*	10.337	1.020-104.759	0.048*
Hydrocephalus	15 (21.7)	16 (27.6)	0.788	0.427–1.453	0.445			
Hyponatremia	16 (23.2)	5 (8.6)	2.690	1.049–6.897	0.028*	0.823	0.077–8.824	0.872
DKA/HHS	14 (20.3)	5 (8.6)	2.354	0.902–1.006	0.066			
Brain abscess	11 (15.9)	6 (10.3)	1.541	0.607–3.911	0.356			
Ischemic infarct	11 (15.9)	4 (6.9)	2.312	0.777–6.874	0.116			
Liver abscess	6 (8.7)	5 (8.6)	1.009	0.324–3.136	0.988			
Vasculitis	7 (10.1)	3 (5.2)	1.961	0.531–7.245	0.300			
Subdural empyema	3 (4.3)	1 (1.7)	2.522	0.270-23.595	0.399			
Spinal abscess	1 (1.4)	3 (5.2)	0.280	0.030–2.622	0.231			
Infective endocarditis	2 (2.9)	2 (3.4)	0.841	0.122–5.783	0.860			
Bacteremia	37 (53.6)	20 (34.5)	1.555	1.025–2.360	0.031*	0.373	0.051–2.716	0.330
Cerebrospinal fluid								
Median (IQR) Glucose level (mmol/L)	1.67 (0.28, 4.58)	1.39 (0.28, 3.69)			0.181			
Median (IQR) Total protein level (g/L)	5.07 (2.36, 7.85)	2.71 (1.44, 4.23)			< 0.001*		0.994–1.001	0.110
Median (IQR) Lactate level (mmol/L)	13.97 (8.55, 18.43)	11.54 (6.72, 19.09)			0.890			
Median (IQR) WBC counts (10^9^/L)	1.07 (0.25, 5.38)	0.72 (0.09, 1.96)			0.827			

OR = odds ratio; CI = confidence interval; mRS: modified Rankin Scale; DKA: diabetic ketoacidosis; HHS: hyperosmolar hyperglycemic state; ^*^ = *p* < 0.05; IQR: interquartile range

a: The ORs and 95% CIs were calculated using univariate logistic regression

b: The ORs and *p* values were calculated using multiple logistic regression

### Implicated pathogens

Table 2 shows the implicated pathogens in the fulminant and non-fulminant groups. *Klebsiella* (*K.*) *pneumoniae* was the most common implicated pathogen in both groups, accounting for 47.8% (33/69) and 29.3% (17/58) of the patients, respectively, and the difference of its infectious rates between the two groups of patients was significant (*p* = 0.040). Of the 50 implicated *K. pneumoniae* strains, 39 strains were identified in the study period of 2000–2009 and the other 11 in the 2010–2022. The 11 *K. pneumoniae* strains isolated in the 2nd study period were found in the time period of 2010–2019 and none was found in the study period of 2020–2022. The results of the antibiotic susceptibility test of the 50 implicated *K. pneumoniae* strains showed that they were all susceptible to both ceftazidime and ceftriaxone. The other implicated pathogens shown in the Table 2 did not show significant difference between the two groups of the enrolled patients.

**Table 2:** The implicated pathogens of the 127 community-acquired spontaneous adult bacterial meningitis patients with a fulminant and non-fulminant clinical course

Pathogen	With fulminant course (N = 69)	With non-fulminant course (N = 58)	*p* value
Gram-negative			
*Klebsiella pneumoniae*	33	17	0.040*
*Pseudomonas aeruginosa*	1	2	0.492
Other *Pseudomonas species*	2	2	0.906
*Escherichia coli*	3	4	0.325
*Neisseria meningitidis*	1	1	0.934
*Proteus mirabilis*	0	1	0.287
*Salmonella enterica serogroup B*	0	1	0.287
*Salmonella enterica serogroup D*	0	1	0.287
*Chryseobacterium meningosepticum*	1	0	0.343
*Sphingomonas paucimobilis*	1	0	0.343
*Roseomonas mucosa*	1	0	0.343
*Fusobacterium nucleatum*	0	1	0.287
Gram-positive			
*Staphylococcus aureus*	6	5	0.638
Coagulase-negative staphylococci	1	2	0.492
*Streptococcus pneumoniae*	6	5	0.908
Viridans streptococci	5	8	0.153
*β-streptococcus group B*	1	1	0.934
*Listeria monocytogenes*	4	4	0.865
*Enterococcus faecalis*	3	0	0.343
Mixed infection	0	3	0.063

The statistical analysis was conducted using univariate analysis; ^*^: *p* < 0.05

### Analysis of prognostic factors

The clinical and laboratory features between the survivors of the fulminant and non-fulminant groups are listed in Table 3. The potential different factors were initial consciousness level, outcomes at discharge, ESRD, immunocompromised state, seizures, DKA/HHS, hyponatremia, septic shock and CSF total protein level. But after multiple logistic regression analysis, none of them were significant. Analysis of the prognostic factors in the 35 patients who died and the other 34 who survived in the fulminant group is shown in Table 4. The results showed that the presence of *K. pneumoniae* infection was a significant factor. Of the 35 patients who died, 13 (37.1%) died ≤ 72 h after symptom onset, and the other 22 (62.9%) died > 72 h after symptom onset. A comparison of the clinical and laboratory features of these two groups of patient is listed in Table 5. The results showed that the presence of ESRD and DKA/HHS were potential factors, however they were all not significant in multiple logistic regression analysis and we found the appropriateness use of antibiotics had no influence of mortality in fulminant CASBM.

**Table 3:** The clinical and therapeutic outcome comparison between the survivors of the community-acquired spontaneous adult bacterial meningitis patients with and without a fulminant clinical course

Characteristic	With fulminant course N = 34 (%)	With non-fulminant course N = 42 (%)	OR^a^	95% CI^a^	*p*-value	OR^b^	95% CI^b^	Adjusted *p*-value
Age	56.32 ± 16.95	55.29 ± 15.73			0.783			
Gender								
Male	24 (70.6)	30 (71.4)	1.042	0.385–2.821	0.936			
Female	10 (29.4)	12 (28.6)						
Initial consciousness level (Glasgow coma scale)	6.21 ± 1.43	13.07 ± 2.51			< 0.001*	-	-	0.984
Outcome (mRS)								
At discharge	3.09 ± 1.73	1.81 ± 1.73			0.002*	-	-	0.991
Three months later	2.56 ± 2.03	1.50 ± 1.85			0.020*	-	-	0.988
Underlying condition								
Diabetes mellitus	20 (58.8)	17 (40.5)	1.453	0.915–2.307	0.112			
Systemic malignancy	5 (14.7)	7 (16.7)	0.882	0.307–2.534	0.816			
Liver cirrhosis	7 (20.6)	3 (7.1)	2.882	0.806–10.312	0.085			
Intracranial hemorrhage	3 (8.8)	10 (23.8)	0.371	0.111–1.241	0.085			
Alcoholism	5 (14.7)	4 (9.5)	1.544	0.449–5.307	0.487			
End-stage renal disease	4 (11.8)	0 (0.0)	1.134	1.002–1.282	0.022*	-	-	0.998
Substance abuser	1 (2.9)	2 (4.8)	0.618	0.058–6.525	0.685			
Brain tumor	1 (2.9)	1 (2.4)	1.235	0.080-19.029	0.879			
Immunocompromised state	18 (52.9)	12 (28.6)	1.853	1.044–3.289	0.031*	-	-	0.999
Clinical manifestations								
Fever	30 (88.2)	37 (88.1)	1.002	0.849–1.182	0.985			
Seizure	17 (50.0)	5 (11.9)	4.200	1.727–10.214	< 0.001*	-	-	1.000
Hydrocephalus	7 (20.6)	10 (23.8)	0.865	0.368–2.030	0.738			
DKA/HHS	9 (26.5)	4 (9.5)	2.779	0.937–8.246	0.051			
Hyponatremia	10 (29.4)	1 (2.4)	12.353	1.663–91.760	0.001*	-	-	0.999
Brain abscess	7 (20.6)	4 (9.5)	2.162	0.690–6.774	0.173			
Septic shock	10 (29.4)	0 (0.0)	1.416	1.140–1.761	< 0.001*	-	-	1.000
Ischemic infarct	7 (20.6)	3 (7.1)	2.882	0.802–10.312	0.085			
Liver abscess	3 (8.8)	3 (7.1)	1.235	0.266–5.7341	0.787			
Vasculitis	4 (11.8)	3 (7.1)	1.647	0.395–6.862	0.488			
Subdural empyema	3 (8.8)	1 (2.4)	3.706	0.403–34.038	0.211			
Spinal abscess	0 (0.0)	2 (4.8)	0.952	0.890–1.019	0.197			
Infective endocarditis	1 (2.9)	1 (2.4)	1.235	0.080-19.029	0.879			
Bacteremia	17 (50.0)	12 (28.6)	1.750	0.975–3.140	0.056			
Cerebrospinal fluid								
Median (IQR) Glucose level (mmol/L)	1.67 (0.28, 6.60)	1.94 (0.28, 3.97)			0.180			
Median (IQR) Total protein level (g/L)	6.15 (2.01, 7.69)	2.66 (1.36, 4.65)			0.012*	-	-	0.992
Median (IQR) Lactate level (mmol/L)	13.54 (8.66, 17.76)	10.55 (6.74, 21.84)			0.796			
Median (IQR) WBC counts (10^9^/L)	1.25 (0.20, 6.67)	0.73 (0.08, 1.84)			0.911			

OR = odds ratio; CI = confidence interval; mRS: modified Rankin Scale; DKA: diabetic ketoacidosis; HHS: hyperosmolar hyperglycemic state; ^*^ = *p* < 0.05; IQR: interquartile range

a: The ORs and 95% CIs were calculated using univariate logistic regression

b: The ORs and *p* values were calculated using multiple logistic regression

**Table 4:** The prognostic factors of the community-acquired spontaneous adult bacterial meningitis patients with a fulminant clinical course

Characteristic	Mortality N = 35 (%)	Survival N = 34 (%)	OR^a^	95% CI^a^	*p*-value
Age	60.34 ± 13.58	56.32 ± 16.95			0.280
Gender					
Male	23 (65.7)	24 (70.6)	1.252	0.454–3.457	0.664
Female	12 (34.3)	10 (29.4)			
Initial consciousness level (Glasgow coma scale)	5.71 ± 1.93	6.21 ± 1.43			0.234
Inappropriate antibiotics use	12 (34.3)	12 (35.3)	1.045	0.388–2.816	0.930
Underlying condition					
Diabetes mellitus	14 (40.0)	20 (58.8)	0.680	0.415–1.114	0.118
Liver cirrhosis	9 (25.7)	7 (20.6)	1.249	0.524–2.974	0.614
Alcoholism	10 (28.6)	5 (14.7)	1.943	0.741–5.096	0.163
End-stage renal disease	6 (17.1)	4 (11.8)	1.457	0.451–4.713	0.526
Systemic malignancy	4 (11.4)	5 (14.7)	0.777	0.228–2.651	0.686
Intracranial hemorrhage	4 (11.4)	3 (8.8)	1.295	0.313–5.362	0.720
Substance abuser	2 (5.7)	1 (2.9)	1.943	0.185–20.446	0.572
Brain tumor	0 (0.0)	1 (2.9)	0.971	0.916–1.029	0.307
Immunocompromised state	23 (65.7)	18 (52.9)	1.241	0.834–1.846	0.280
Clinical manifestations					
Initial altered consciousness	32 (91.4)	31 (91.2)	1.003	0.867–1.160	0.970
Fever	29 (82.9)	30 (88.2)	0.939	0.773–1.140	0.526
Seizure	15 (42.9)	17 (50.0)	0.857	0.515–1.426	0.552
Septic shock	18 (51.4)	10 (29.4)	1.749	0.948–3.2251	0.063
Hyponatremia	6 (17.1)	10 (29.4)	0.583	0.238–1.427	0.227
Hydrocephalus	8 (22.9)	7 (20.6)	1.110	0.452–2.725	0.819
DKA/HHS	5 (14.3)	9 (26.5)	0.540	0.201–1.447	0.208
Brain abscess	4 (11.4)	7 (20.6)	0.555	0.179–1.726	0.299
Ischemic infarct	4 (11.4)	7 (20.6)	0.555	0.179–1.726	0.299
Vasculitis	3 (8.6)	4 (11.8)	0.729	0.176–3.016	0.660
Liver abscess	3 (8.6)	3 (8.8)	0.971	0.211–4.482	0.970
Infective endocarditis	1 (2.9)	1 (2.9)	0.971	0.063-14.9151	0.983
Bacteremia	20 (57.1)	17 (50.0)	1.143	0.735–1.778	0.552
* K. pneumoniae* infection	22 (62.9)	11 (32.4)	1.943	1.122–3.363	0.011*
Cerebrospinal fluid					
Median (IQR) Glucose level (mmol/L)	1.64 (0.28, 3.97)	1.67 (0.28, 6.60)			0.211
Median (IQR) Total protein level (g/L)	4.95 (2.71, 8.32)	6.15 (2.01, 7.69)			0.893
Median (IQR) Lactate level (mmol/L)	14.65 (7.22, 18.87)	13.54 (8.66, 17.76)			0.673
Median (IQR) WBC counts (10^9^/L)	0.60 (0.28, 4.26)	1.25 (0.20, 6.67)			0.287

OR = odds ratio; CI = confidence interval; DKA: diabetic ketoacidosis; HHS: hyperosmolar hyperglycemic state; *K. pneumoniae*: *Klebsiella pneumoniae*; ^*^ = *p* < 0.05; IQR: interquartile range

a: The ORs and 95% CIs were calculated using univariate logistic regression

**Table 5:** The affecting factors of the time of mortality in the community-acquired spontaneous adult bacterial meningitis patients with a fulminant clinical course

Characteristic	≤ 72 h N = 13 (%)	> 72 h N = 22 (%)	OR^a^	95% CI^a^	*p*-value	OR^b^	95% CI^b^	Adjusted *p*-value
Age	62.31 ± 14.55	59.18 ± 13.19			0.519			
Gender								
Male	10 (76.9)	13 (59.1)	0.433	0.092–2.031	0.283			
Female	3 (23.1)	9 (40.9)						
Initial consciousness level (Glasgow coma scale)	5.69 ± 2.10	5.73 ± 1.88			0.960			
Inappropriate antibiotics use	5 (38.5)	7 (31.8)	1.862	0.203-2.600	0.840			
Underlying condition								
Diabetes mellitus	6 (46.2)	8 (36.4)	1.269	0.567–2.843	0.568			
Alcoholism	4 (30.8)	6 (27.3)	1.128	0.390–3.267	0.825			
Liver cirrhosis	2 (15.4)	7 (31.8)	0.484	0.118–1.988	0.282			
Systemic malignancy	2 (15.4)	2 (9.1)	1.692	0.270-10.614	0.572			
Intracranial hemorrhage	1 (7.7)	3 (13.6)	0.564	0.065–4.876	0.593			
End-stage renal disease	0 (0.0)	6 (27.3)	0.729	0.563–0.939	0.039*	-	-	0.999
Substance abuser	0 (0.0)	2 (9.1)	0.909	0.797–1.037	0.263			
Immunocompromised state	8 (61.5)	15 (68.2)	0.903	0.539–1.512	0.689			
Clinical manifestations								
Fever	10 (76.9)	19 (86.4)	0.891	0.633–1.253	0.474			
Septic shock	7 (53.8)	11 (50.0)	1.077	0.560–2.071	0.8261			
Seizure	6 (46.2)	9 (40.9)	1.128	0.521–2.4432	0.762			
Hydrocephalus	2 (15.4)	6 (27.3)	0.564	0.133–2.395	0.418			
DKA/HHS	4 (30.1)	1 (4.5)	6.769	1.845–54.253	0.032*	-	-	0.112
Hyponatremia	3 (23.1)	3 (13.6)	1.692	0.399–7.186	0.474			
Brain abscess	1 (7.7)	3 (13.6)	0.564	0.065–4.876	0.593			
Ischemic infarct	2 (15.4)	2 (9.1)	1.692	0.270-10.614	0.572			
Liver abscess	1 (7.7)	2 (9.1)	0.846	0.085–8.444	0.886			
Vasculitis	1 (7.7)	2 (9.1)	0.846	0.085–8.444	0.886			
Bacteremia	9 (69.2)	11 (50.0)	1.385	0.796–2.407	0.267			
* K. pneumoniae* infection	11 (84.6)	11 (50.0)	5.000	0.884–28.288	0.056			
Cerebrospinal fluid								
Median (IQR) Glucose level (mmol/L)	2.69 (0.76, 5.67)	0.44 (0.28, 3.89)			0.114			
Median (IQR) Total protein level (g/L)	7.13 (2.60, 1.17)	4.64 (2.04, 7.72)			0.146			
Median (IQR) Lactate level (mmol/L)	17.76 (11.29, 22.76)	12.32 (5.86, 17.59)			0.089			
Median (IQR) WBC counts (10^9^/L)	0.60 (0.33, 4.41)	1.47 (0.27, 5.37)			0.332			

OR = odds ratio; CI = confidence interval; DKA: diabetic ketoacidosis; HHS: hyperosmolar hyperglycemic state; ^*^ = *p* < 0.05; IQR: interquartile range

a: The ORs and 95% CIs were calculated by using univariate logistic regression

b: The ORs and *p* values were calculated by using multiple logistic regression

## Discussion

Many factors including age, underlying medical/surgical conditions, vaccination status and bacterial pathogens may influence the clinical manifestations, course and therapeutic outcomes of adult bacterial meningitis [1–5, 16]. Clinically, we defined fulminant bacterial meningitis as a syndrome consisted of the following pictures: a sudden onset of symptomatic meningitis, a rapid deterioration of neurological signs with or without shock, abrupt cerebral edema, or intractable intracranial hypertension [17]. The pathophysiology and pathogenesis of bacterial meningitis involved complicated interaction between pathogen factors and host immune response. Meningeal pathogens also increase the permeability of the blood-brain barrier (BBB), allowing pathogens and cytokines and neutrophils move into the subarachnoid space and the intense subarachnoid inflammatory response leads to consequences of bacterial meningitis including cerebral edema and increased intracranial pressure [18]. For the high mortality and morbidity in bacterial meningitis without appropriate treatment, we analyzed clinical presentations and prognostic factors in patients with or without fulminant course of bacterial meningitis and tried to uncover the red flag of the fulminant bacterial meningitis. In the 23-year study period, spontaneous adult bacterial meningitis accounted for 31.1% (139/447) of the overall adult bacterial meningitis cases, and the other 68.9% (308/447) were caused by post-neurosurgical infections. This relative relatively lower rate of spontaneous infections and higher rate of post-neurosurgical infections is consistent with our previous epidemiologic studies of adult bacterial meningitis in Taiwan [1, 5, 19]. Of the enrolled 139 patients with adult bacterial meningitis with spontaneous infections, 91.4% (127/139) had community-acquired infections. Of these 127 patients, 54.3% (69/127) had a fulminant clinical course and the other 45.7% (58/127) had a non-fulminant clinical course.

As shown in Table 1, the CASBM patients with ESRD as the preceding event had a significantly higher rate of developing a fulminant clinical course, and those with a fulminant clinical course also had significantly more severe clinical presentations including initial presentation of altered consciousness, septic shock, seizures, and a higher CSF total protein level. In addition, compared to those without a fulminant clinical course, those with a fulminant clinical course had a higher mortality rate (50.7%, 35/69) (Table 1), and this figure of mortality rate was much higher than that of the overall group of patients with ABM (25.5%) [1]. It is well known that ESRD is associated with an increased risk of infection and infection-related mortality [19, 20]. In Taiwan, the number of patients receiving maintenance dialysis is increasing rapidly, and Taiwan now has the highest incidence of ESRD globally [21]. The higher incidence of ESRD and severe neurologic manifestations of the CASBM patients with a fulminant clinical course (Table 1) are known to be important prognostic factors of ABM [22–25]. In addition, those who had a fulminant course and survived had significantly worse therapeutic outcomes at discharge and at 3 months after discharge. The significant factors associated with the poor therapeutic outcomes included initial consciousness level and seizures (Table 3); both of which are known to be important prognostic factors of bacterial meningitis [1, 26].

In contrast to the epidemiologic trend of bacterial meningitis in Western countries, in which *Streptococcus* (*S*.) *pneumoniae* is the most common and important bacterial pathogen of community-acquired bacterial meningitis [27, 28], *K. pneumoniae* is the most implicated pathogen of meningitis in Taiwan [1, 5, 25]. Because of the vaccination program in Taiwan, the incidence of *S*. *pneumoniae* infection in adult bacterial meningitis has decreased gradually [1]. The annual incidence of overall *S. pneumoniae* infection decreased from 41.2 per 1000 to 15.2 per 1000 in children and from 5.0 per 1000 to 1.5 per 1000 in adult from a 12-year-period study review and in this study, the meningitis caused by *S. pneumoniae* accounted for 1.3% of all adult patient with *S. pneumoniae* infection [29]. From studies published between 2010 and 2019, the annual incidence of *S. pneumoniae* meningitis was around 24.3% in India, 6.4% in Singapore, 3.1% in Korea and 0.2–0.26 per 100,000 population in Japan [30–33]. The above data were comparable with the result of higher incidence was published in India and lower in Taiwan and Japan [34]. Although a post-neurosurgical state is the preceding event in some patients with adult *K. pneumoniae* meningitis, *K. pneumoniae* meningitis is usually acquired spontaneously in the community. Therefore, it is not surprising that *K. pneumonia*e was the most common pathogen in the 127 enrolled CASBM patients, accounting for 40.3% (50/124) of those with a monomicrobial infection (Table 2). Other than *K. pneumonia*, several other implicated bacterial pathogens were found in the 127 patients (Table 2), however only the presence of *K. pneumoniae* infection was a significant factor for the development of a fulminant clinical course. Even though *K. pneumoniae* strains have been reported to have a high level of carbapenem resistance and broad resistance to many beta-lactam antibiotics in Taiwan [35, 36], none of the 50 enrolled *K. pneumoniae* strains showed resistance to either ceftriaxone or ceftazidime. Both of these cephalosporins are commonly used as empiric antibiotics for the treatment of adult bacterial meningitis in Taiwan.

In this study, more than half of the CASBM patients with a fulminant clinical course died (Tables 1 and 4). As shown in Table 4, the presence of *K. pneumoniae* infection was the most important factor for mortality in this specific group of patients. *K. pneumoniae* infection including meningitis is a very distinctive infectious syndrome in Taiwan [37–40]. Many factors may influence the therapeutic results of *K. pneumoniae* meningitis, however the timing of appropriate antimicrobial therapy is currently the major determinant of survival and neurological outcomes for this group [41, 42]. The high mortality rate of the CASBM patients with a fulminant clinical course may be related to the rapid deterioration in consciousness before the use of appropriate antimicrobial agents. Besides those with *K. pneumoniae* infection, the case numbers of the other implicated pathogens in the patients with this specific infectious syndrome were too small to allow for adequate analysis. Both DKA/HHS and ESRD are severe medical conditions which may make the patients vulnerable to infectious diseases and poor therapeutic outcomes [12, 15–17]. However, although both factors were potentially associated with the development of early mortality (≤ 72 h) in the 35 patients with CASBM and a fulminant course who died, neither factor was significant in multiple logistic regression analysis (Table 5). As shown in our previous study [43], we found higher mortality in ESRD patients with CASBM and the most implicated pathogen was *S. aureus*, through the catheter for dialysis and higher resistant rate to penicillin and oxacillin were noticed in *S. aureus*. Therefore, the physicians should choose vancomycin rather than oxacillin as the empirical antibiotics for CASBM patients under dialysis.

From the result of this study, we found high mortality rate of this group of CASBM patients; therefore, aggressive medical support and timely using appropriate antibiotics were crucial for the clinicians in treating this specific group of infectious disease. However, it needs time for the culture result available, so the more rapid test for pathogen detection is necessary. In addition to the above-mentioned appropriate antibiotics use, early detection of pathogens and the susceptibility of antibiotics by using polymerase chain reaction (PCR) test [44, 45] may be also important for a successful treatment of fulminant CASBM.

### Limitations

There are several limitations to this study: (1) patients with culture negative ABM were not included in the study, and (2) the choice of GSC score < 8 to define the fulminant clinical course may have led to bias influencing patient group classification and the analysis of the prognostic factors.

## Conclusions

In the present study, 54.3% of the CASBM patients had a fulminant clinical course, and the presence of ESRD was an important factor for its development. This specific group of patients had severe neurologic manifestations and also a high mortality rate (50.7%). In Taiwan, *K. pneumoniae* is an important implicated pathogen of adult bacterial meningitis, especially the community-acquired spontaneous form. It was also an important factor for the development of CASBM with a fulminant clinical course and the high mortality rate of the patients in this study. In addition, of the patients with CASBM and a fulminant clinical course who died, 18.8% died ≤ 72 h after symptom onset. In addition, the CASBM patients with a fulminant clinical course who survived had poor therapeutic outcomes at discharge and at 3 months after discharge.

## Data Availability

The datasets used and/or analyzed during the current study available from the first author and the corresponding author on reasonable request.

## Abbreviations

CASBM: Community acquired spontaneous bacterial meningitis
CNS: Central nervous system
CSF: Cerebrospinal fluid
DKA: Diabetic ketoacidosis
ESRD: End-stage renal disease
HHS: Hyperosmolar hyperglycemic state
mRS: Modified Rankin scale
